# Totally laparoscopic excision of a retroperitoneal lipoma extending into the inguinal canal: a rare case report with intraoperative video

**DOI:** 10.1097/RC9.0000000000000637

**Published:** 2026-07-08

**Authors:** Yoshihisa Okuchi, Meiki Fukuda, Takaaki Yakushigawa, Takayuki Kawai, Kojiro Taura, Hiroaki Terajima

**Affiliations:** Department of Gastroenterological Surgery and Oncology, Kitano Hospital Medical Research Institute, Osaka, Japan

**Keywords:** case report, inguinal hernia, internal inguinal ring, laparoscopic surgery, retroperitoneal lipoma, round ligament

## Abstract

**Introduction:**

Retroperitoneal lipoma extending into the inguinal canal is extremely rare. Its distinction from well-differentiated liposarcoma can be challenging. This report describes the first case of a totally laparoscopic intraperitoneal excision and highlights the anatomical considerations necessary for a safe resection.

**Presentation of case:**

A 58-year-old woman presented with a long-standing, non-reducible right inguinal bulge. Magnetic resonance imaging and computed tomography revealed a homogeneous lipomatous mass arising at the right pelvic inlet, protruding through the internal inguinal ring, and compressing the bladder and cecum without invasion. Laparoscopic resection was performed under general anesthesia using five ports. The tumor was dissected carefully along the external iliac vessels. The round ligament was divided to mobilize the mass from the inguinal canal. The enlarged internal inguinal ring was closed with continuous barbed suturing. Mesh placement was avoided in consideration of the benign characteristics of the lesion. The specimen measured 13.5 × 11.0 × 3.0 cm. Histopathology confirmed mature adipose tissue negative for MDM2 and CDK4. Recovery was uneventful. The patient was discharged on postoperative day 4.

**Discussion:**

A totally laparoscopic intraperitoneal approach provides excellent visualization of pelvic anatomy, thereby facilitating safe dissection and anatomical repair with minimal invasiveness. This technique might be a less invasive alternative to open surgery when malignancy is not suspected.

**Conclusion:**

This case suggests a totally laparoscopic approach as a feasible, minimally invasive option for treating a retroperitoneal lipoma with inguinal extension.

## Introduction

Lipomas, benign tumors composed of mature adipocytes, most commonly arise in the trunk or extremities[1]. Their occurrence in the retroperitoneum is extremely rare. Retroperitoneal tumors themselves are uncommon, accounting for approximately 0.2% of all neoplasms[2]. About 70–80% of these tumors are reported as malignant^[^3,4^]^. Therefore, when managing retroperitoneal tumors, the possibility of malignancy must always be considered.

HIGHLIGHTS

- Retroperitoneal lipoma extending into the inguinal canal is extremely rare.
- This report is the first to describe its totally laparoscopic intraperitoneal excision.
- Dissection requires anatomical details of the inguinal canal and the round ligament.
- Minimally invasive surgery can treat retroperitoneal tumors with groin extension.

Retroperitoneal lipoma represents around 3.0% of retroperitoneal tumors; it is an exceedingly rare entity^[^4,5^]^. Differentiation from well-differentiated liposarcoma (WDLS) based on imaging findings is particularly challenging, especially in large tumors or those showing gradual enlargement over time. Careful diagnostic evaluation is, therefore, extremely important in such cases.

We report herein an extremely rare case of a retroperitoneal lipoma that arose in the pelvic cavity and partially herniated through the inguinal canal, presenting as a groin bulge mimicking an inguinal hernia. The tumor was completely resected laparoscopically via an intraperitoneal approach. The associated clinical characteristics, surgical findings, and relevant literature are discussed.

This case report has been reported in line with the SCARE checklist[6].

## Presentation of case

A 58-year-old woman (body mass index: 17.6 kg/m^2^) was referred to our department for evaluation of a right pelvic mass that had been detected incidentally by her gynecologist during a routine examination. Fifteen years earlier, she had been diagnosed with a right inguinal hernia after noticing swelling in the groin. However, because she experienced neither pain nor discomfort, her case was managed conservatively. On physical examination, the abdomen was soft; a firm, elastic mass of approximately 4 cm in diameter was palpable in the right inguinal region. A slight bulge was also visible when she was in the supine position. The mass was non-reducible. Routine laboratory findings were unremarkable.

**Figure 1.**
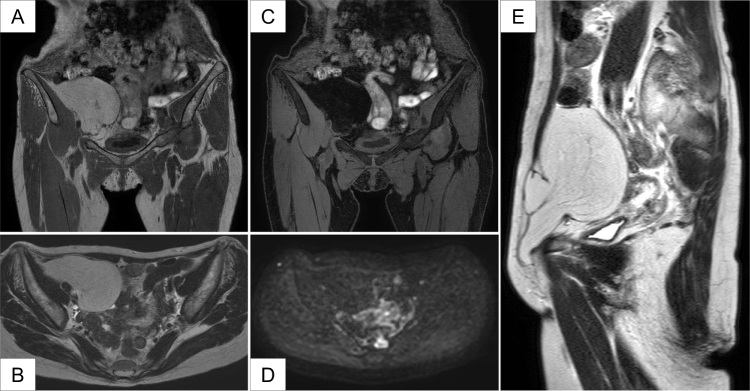
Coronal T1-weighted (A) and axial T2-weighted (B) images show a retroperitoneal mass with high signal intensity, similar to subcutaneous fat. On the coronal fat-suppressed T1-weighted image (C), the signal is uniformly low, which is consistent with a fatty lesion. The axial diffusion-weighted image (D) shows no restricted diffusion. The sagittal T2-weighted image (E) shows the tumor protruding into the right inguinal region.

**Figure 2.**
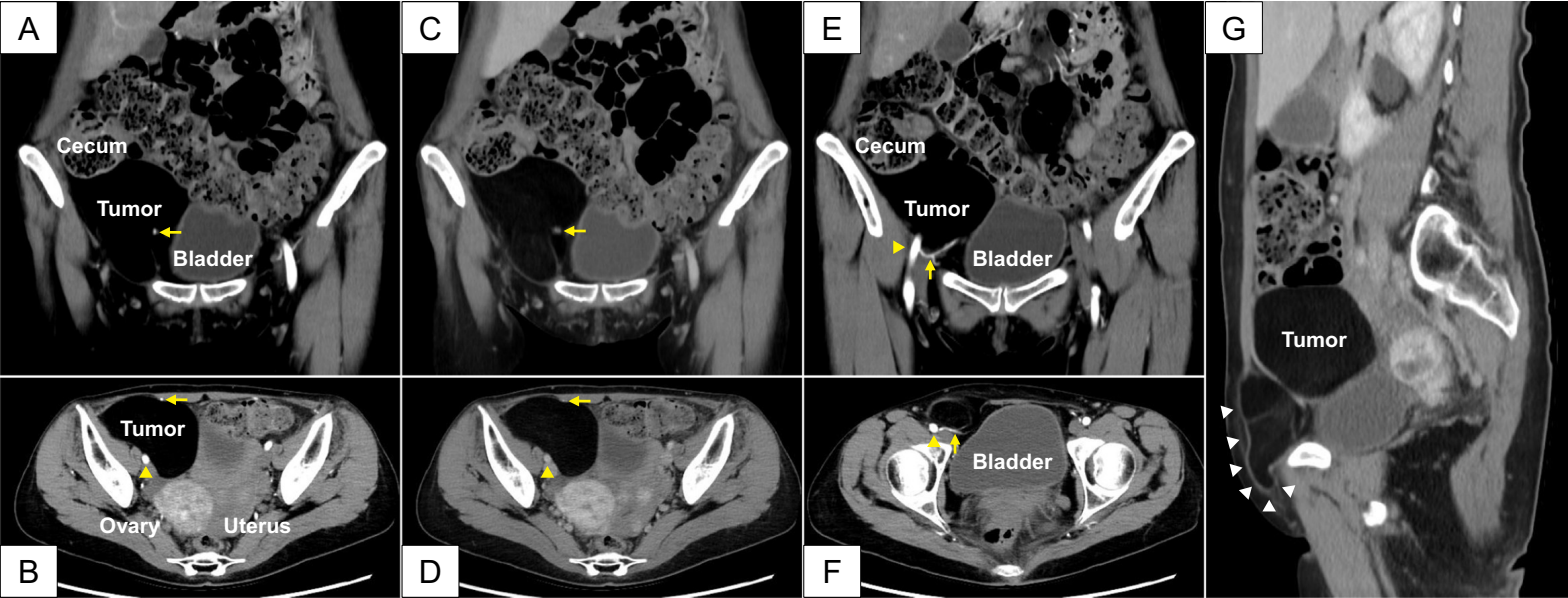
Contrast-enhanced CT findings of the pelvic mass. Coronal (A) and axial (B) images in the early phase, and coronal (C) and axial (D) images in the delayed phase show a well-defined retroperitoneal mass located anterior to the external iliac vessels, compressing the adjacent structures without internal enhancement. Early-phase coronal (E) and axial (F) images show the inferior epigastric vessels located caudal to the tumor. Yellow arrowheads and arrows, respectively, indicate the right external iliac artery and the inferior epigastric artery. White arrowheads in (G) indicate the tumor prolapsing into the inguinal canal.

Pelvic magnetic resonance imaging (MRI) revealed a retroperitoneal mass measuring over 10 cm at the right pelvic inlet, protruding into the right inguinal region. The lesion exhibited high signal intensity on both T1-weighted and T2-weighted images (T1WI and T2WI) and low signal intensity on fat-suppressed T1WI, findings consistent with a fatty composition. No high signal was observed on diffusion-weighted images (Fig. 1). Contrast-enhanced computed tomography (CT) demonstrated compression of the cecum, bladder, and uterus, but no signs of direct invasion into adjacent structures. The tumor was located cranial to the right external iliac vessels, with compression of the inferior epigastric vessels (Fig. 2). Abdominal ultrasonography revealed no internal vascularity. These findings were consistent with a retroperitoneal tumor herniating through the inguinal canal.

Based on the imaging findings, a benign retroperitoneal lipoma was suspected. Nevertheless, the possibility of a WDLS could not be ruled out completely. After obtaining informed consent, surgical resection was planned using a laparoscopic approach under general anesthesia. The patient was placed in the Trendelenburg position. A 12-mm camera port was introduced at the umbilicus using the open technique. Two additional ports (5 and 12 mm) were placed as operator ports along the left midclavicular line. Another 5-mm port was inserted in the right upper abdomen for assistance. An additional 5-mm assistant port was later placed in the right lower abdomen (Supplemental Digital Content Figure 1, available at: http://links.lww.com/IJSCR/A84).

**Figure 3.**
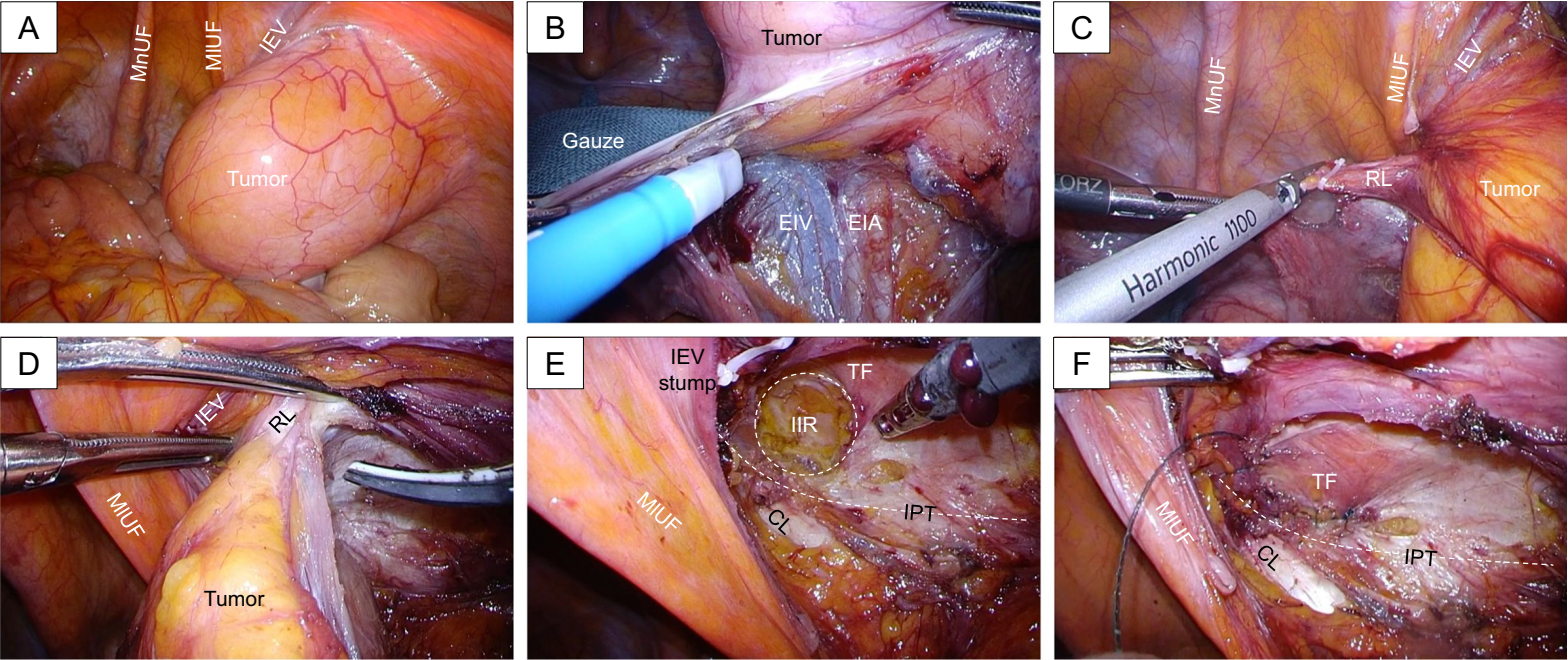
Intraoperative findings. (A) Overall view of the tumor in the right inguinal region. (B) Dissection between the tumor and the external iliac vessels on the dorsal side of the tumor. The material labeled “Gauze” is surgical gauze, not prosthetic mesh. (C) Division of the proximal end of the round ligament. (D) The tumor was gently retracted anteriorly and dissected while being pulled out from the internal inguinal ring. (E) Overview of the widened internal inguinal ring after tumor resection. (F) Closure of the internal inguinal ring by continuous barbed suturing. MnUF: median umbilical fold; MlUF: medial umbilical fold; IEV: inferior epigastric vessels; EIV: external iliac vein; EIA: external iliac artery; RL: round ligament; IIR: internal inguinal ring; CL: Cooper ligament; IPT: iliopubic tract; TF: transversalis fascia.

The tumor, which was located in the retroperitoneal space of the right inguinal region, protruded into the peritoneal cavity (Fig. 3A). It appeared to be well demarcated from adjacent structures. First, the mass was dissected carefully along the external iliac vessels (Fig. 3B). Where the tumor adhered to retroperitoneal tissue, a thin layer of surrounding fat was excised *en bloc* with the specimen. The round ligament was divided (Fig. 3C). The inferior epigastric vessels, which were injured inadvertently, were subsequently divided as well. The tumor was gently retracted anteriorly during dissection from the inguinal canal. The distal round ligament was divided (Fig. 3D). After tumor removal, the enlarged internal inguinal ring was closed with continuous suturing to restore the defect (Fig. 3E and 3F). The specimen was enclosed in a retrieval bag. The umbilical incision was extended to 48 mm for extracorporeal extraction. An anti-adhesive agent was applied to the peritoneal defect in the right inguinal area. Then, the procedure was completed (Supplemental Digital Content Video 1, available at: http://links.lww.com/IJSCR/A85). The operative time was 176 min, with minimal blood loss. The postoperative course was uneventful. The patient was discharged on postoperative day 4.

The resected specimen measured 13.5 × 11.0 × 3.0 cm (Supplemental Digital Content Figure 2A, available at: http://links.lww.com/IJSCR/A84). Histopathological examination revealed mature adipose tissue composed of uniform adipocytes without cellular atypia (Supplemental Digital Content Figure 2B and C, available at: http://links.lww.com/IJSCR/A84). Immunohistochemical analysis showed negative staining for murine double minute (MDM2) and cyclin-dependent kinase 4 (CDK4), thereby confirming the diagnosis of retroperitoneal lipoma (Supplemental Digital Content Figure 2D and E, available at: http://links.lww.com/IJSCR/A84).

At 6 months after surgery, the patient remained free of recurrence.

## Discussion

This case involves an extremely rare retroperitoneal lipoma, part of which herniated through the internal inguinal ring into the groin. Retroperitoneal lipomas are much less common than liposarcomas, accounting for only a small proportion of primary retroperitoneal tumors. Among reported retroperitoneal lipomas, those extending through the inguinal canal are exceedingly rare, with only six similar cases reported in the literature^[^7–12^]^: three of these cases presented as inguinal-hernia-like protrusions^[^7–9^]^; the remaining three as femoral-hernia-like protrusions^[^10–12^]^. In all reported cases, the tumors were large and protruded through the groin, necessitating an inguinal incision. In many of them, an additional midline or lower abdominal incision was also made. We performed a literature search using PubMed and Google Scholar for English-language articles published up to May 2026. The search terms included combinations of “retroperitoneal lipoma,” “inguinal canal,” “inguinal hernia,” “femoral hernia,” “laparoscopic,” “laparoscopy,” “intraperitoneal approach,” and “excision.” Based on this search, to our knowledge, this is the first reported case of complete laparoscopic excision performed entirely via an intraperitoneal approach.

In our patient, preoperative imaging suggested a well-circumscribed, homogeneous fatty mass without evidence of invasion. Malignancy was not strongly suspected. Therefore, we selected a laparoscopic approach to minimize invasiveness and to improve visualization of the pelvic anatomy. The tumor’s softness and mobility also favored safe manipulation under laparoscopy. This case demonstrates that, when malignancy is unlikely and when the tumor is located near the pelvic inlet, laparoscopic surgery may represent a safe and feasible alternative to open resection, offering superior visualization, reduced postoperative pain, and faster recovery.

Because WDLS could not be completely excluded preoperatively, the oncological safety of the laparoscopic approach required careful consideration. The main concerns were tumor rupture, capsular injury, and incomplete resection. During the operation, careful traction and dissection were performed to avoid capsular disruption, and the tumor was removed *en bloc*. Therefore, a laparoscopic approach should be considered only when complete resection without tumor rupture is judged to be feasible.

A precise understanding of the anatomy was crucial to the success of this operation. We anticipated that the tumor had prolapsed through the inguinal canal along the course of the right round ligament. This assumption was confirmed intraoperatively. The round ligament, therefore, served as a useful anatomical landmark. *En bloc* resection, including the round ligament, allowed for safe and complete tumor removal. The dissection proceeded in close proximity to the external iliac vessels and inferior epigastric vessels, both of which were well identified because of the magnified laparoscopic view. After excision, the enlarged internal inguinal ring was closed with continuous barbed suturing between the iliopubic tract and transversalis fascia. Given the benign nature of the lesion and the potential need for reoperation in the event of recurrence, mesh placement was intentionally avoided. This approach provided secure closure and minimized the risk of foreign-body implantation.

From a diagnostic perspective, distinguishing retroperitoneal lipoma from WDLS remains an important challenge. In this case, MRI revealed homogeneous high signal intensity on both T1WI and T2WI, with signal suppression on fat-suppressed sequences, consistent with a lipomatous lesion. Imaging findings that may suggest WDLS include thickened internal septa, nodular or non-adipose components, heterogeneous signal intensity, and enhancement within septa or solid components. In the present case, these suspicious findings were not evident; however, slow enlargement of the mass during a 15-year interval raised concern about possible WDLS, as low-grade liposarcoma may also demonstrate gradual growth. Histopathological examination revealed mature adipose tissue without atypia. Moreover, immunohistochemical staining for MDM2 and CDK4 was negative, supporting the diagnosis of retroperitoneal lipoma. Although fluorescence *in situ* hybridization for MDM2 amplification was not performed due to clear immunohistochemical findings, previous studies have demonstrated its utility in differentiating lipoma from WDLS[13]. Given reports of recurrence even in histologically benign cases[14], careful long-term follow-up is warranted.

The present case also emphasizes the importance of including retroperitoneal lipoma in the differential diagnosis of fatty lesions presenting as inguinal or femoral hernia-like masses. When a non-reducible groin mass lacks a true hernia sac or exhibits atypical imaging features, a retroperitoneal origin should be considered. Awareness of this possibility is essential to prevent inadvertent tumor injury during hernia repair.

## Conclusion

This case highlights the feasibility of a totally laparoscopic approach used for a retroperitoneal lipoma with inguinal extension, provided that preoperative imaging and intraoperative findings indicate a benign lesion. Accumulation of additional laparoscopic cases will help clarify optimal indications and refine surgical strategies for this rare condition.

## Data Availability

All relevant data supporting the conclusions of this case report are included in the article and its supplementary materials. Additional patient-level data are not publicly available due to privacy and ethical restrictions.
